# Low-Molecular-Weight Heparin Use in a Case of Noncardiogenic Multifocal Perinatal Thromboembolic Stroke

**DOI:** 10.1155/2009/153643

**Published:** 2009-02-16

**Authors:** Matthew A. Saxonhouse, Dan Tarquinio, Paul R. Carney, Jeff Bennett, Amy Smith, Stephen P. Hunger, James D. Geyer

**Affiliations:** ^1^Division of Neonatology, Department of Pediatrics, University of Florida, Gainesville, FL 32610-0296, USA; ^2^Division of General Pediatrics, Department of Pediatrics, University of Florida, Gainesville, FL 32610-0296, USA; ^3^Division of Neurology, Department of Pediatrics, University of Florida, Gainesville, FL 32610-0296, USA; ^4^Department of Radiology, University of Florida, Gainesville, FL 32610-0296, USA; ^5^Division of Hematology/Oncology, Department of Pediatrics, University of Florida, Gainesville, FL 32610-0296, USA; ^6^Section of Pediatric Hematology/Oncology/Bone Marrow Transplantation and Center for Cancer and Blood Disorders, The Children's Hospital, University of Colorado, Denver School of Medicine, Aurora, CO 80045, USA; ^7^Division of Neurology and Sleep Medicine, Department of Internal Medicine, College of Community Health Sciences, University of Alabama, Tuscaloosa, AL 35487, USA

## Abstract

A full-term neonate suffered multifocal cerebral infarctions due to multiple large vessel thrombi. Thrombophilia and cardiovascular assessments were negative, but due to the severity of the lesions and the concern for expansion of the thrombi or future embolic events, treatment with low-molecular-weight heparin (LMWH) was initiated. No complications from treatment were experienced. We present this severe case in order to highlight difficult management decisions for newborns with multifocal perinatal thromboembolic stroke and to stress the need for further practice guidelines and research in this area.

## 1. Introduction

Perinatal stroke, occurring between 20 weeks' gestation through the 28th postnatal day [1–3], has a prevalence ranging from 17.0 to 43.4 per 100 000 live births [4–6]. The majority (80%) of these events is ischemic in nature and most abnormalities occur in the left hemisphere within the distribution of the middle cerebral artery (MCA). Rarely, multifocal cerebral infarctions can occur and these tend to be embolic in origin [7, 8].

Despite the prevalence of perinatal stroke, no randomized clinical trials exist to address acute management or prevention [1, 8, 9]. Current guidelines from the American College of Chest Physicians (ACCP) suggest anticoagulation or aspirin therapy only for neonates with a first perinatal stroke with a documented ongoing cardioembolic source (Grade IB) or recurrent arterial ischemic stroke (AIS; Grade 2C) [9]. However, recent recommendations from the American Heart Association (AHA) suggest that anticoagulation may be selected in neonates with multiple cerebral emboli (Class IIb, Level of Evidence C) [1].

We present a neonate who suffered multifocal cerebral infarctions due to thrombosis of five vessels. No evidence suggested cardiac disease or a prothrombotic disorder. Due to the severity, size, and locations of the thrombi, plus the multifocal presentation of the cerebral infarctions, we decided to anticoagulate the patient with low-molecular-weight heparin (LMWH) in an attempt to decrease the risk of extension of the thrombi and/or secondary emboli. This case of multifocal thromboembolic perinatal stroke represents the most severe end of the spectrum and highlights the difficulties inherent in management of these types of patients.

## 2. Case Report

A 40-week, 2884 g, female infant was delivered via cesarean section to a 41-year-old primigravida after arrest of descent and failed vacuum extraction. Complications of the pregnancy included a history of infertility, gestational diabetes (glyburide controlled), prolonged rupture of membranes, and chorioamnionitis. Apgar scores were 1, 7, and 8. Umbilical artery and venous pH were 6.94 and 7.06, respectively. Initial blood testing was nonspecific. Physical examination was significant for a large left caput succedaneum with a soft and flat anterior fontanel. Neurological examination demonstrated mild hypotonia. At approximately 36 hours of age, the patient had seizure activity consisting of eye fluttering, bilateral clonic movements of the upper extremities, and tongue thrusting. Two separate events occurred, each lasting less than three minutes.

Repeat blood testing revealed a normal glucose level, no significant electrolyte abnormalities, a white blood count of 9.8 × 10^3^/mm^3^, and hematocrit of 46.8%. The platelet count had decreased from 155 (initial) to 119 × 10^3^/mm^3^ with subsequent drop to 109 × 10^3^/mm^3^ twenty four hours later. Cerebrospinal fluid evaluation revealed (traumatic lumbar puncture) 115 000/mm^3^ red blood cells, 50/mm^3^ white blood cells with 87% polys and 12% lymphs. The patient was given a loading dose of phenobarbital, and ampicillin and gentamicin had been started shortly after birth.

**Figure 1:**
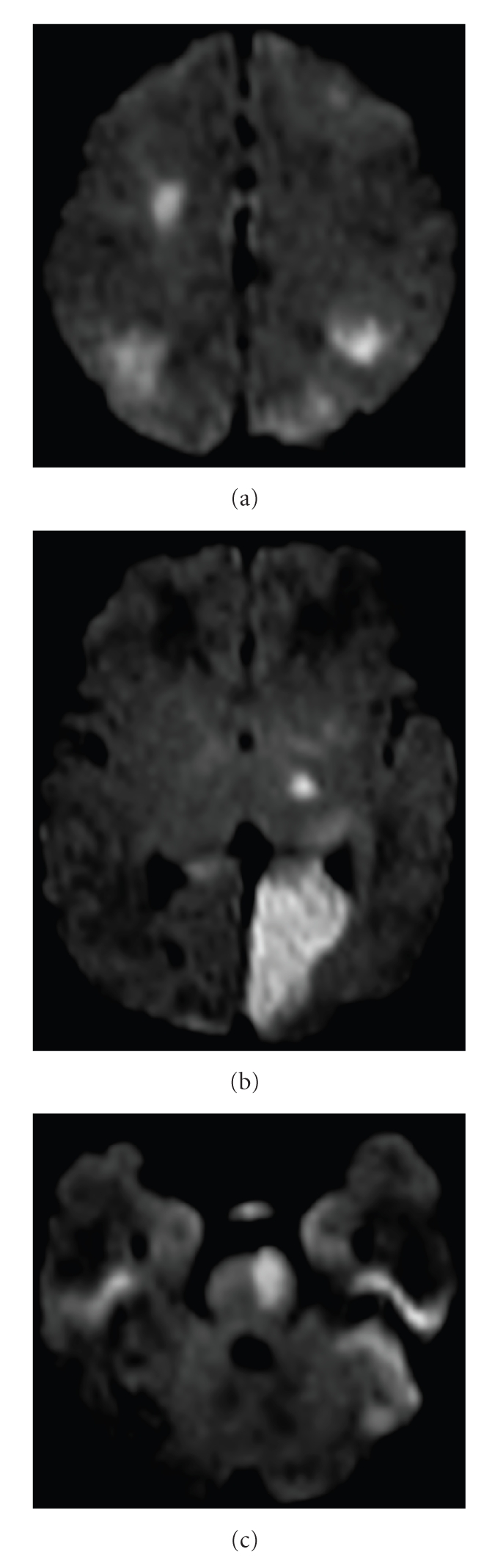
(a)–(c): Axial diffusion-weighted images, *b* = 1000, demonstrate multiple areas of high signal in bilateral frontal and parietal lobes, left occipital lobe, left thalamus, and left pons.

**Figure 2:**
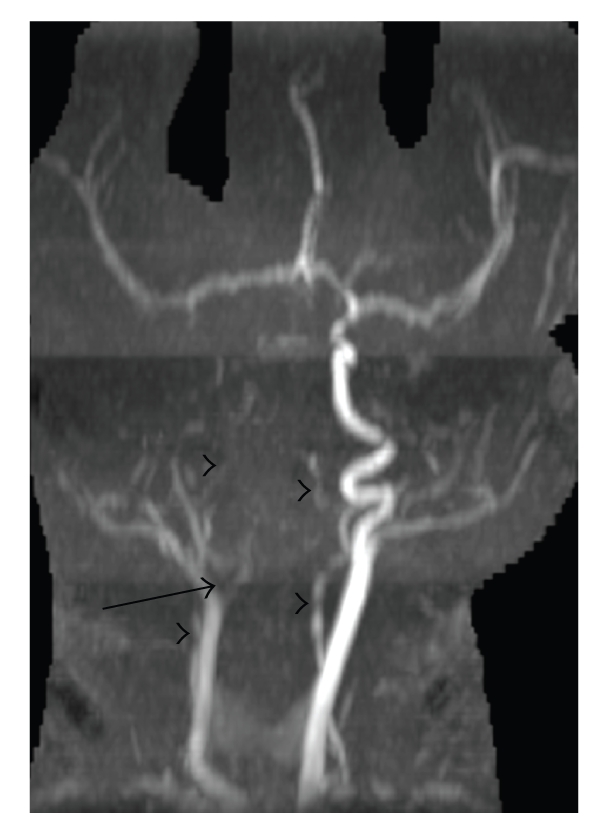
3D maximum-intensity projection (MIP) image from a 3D time of flight MRA demonstrates occlusion of the right internal carotid artery at its origin (arrow). The vertebral arteries are diminutive (arrowheads) and the basilar artery is not visualized.

Magnetic resonance imaging (MRI) of the brain (Figure 1) demonstrated bilateral multifocal regions of increased signal on diffusion-weighted imaging in the left pons, left occipital lobe, left thalamus, left and right frontal lobes, and left and right frontoparietal regions. No evidence indicated hemorrhage, and all of the lesions were dark on apparent diffusion coefficient of water (ADC) mapping indicating that they were acute in nature (within 1 week). A transthoracic echocardiogram demonstrated normal cardiac anatomy with no intracardiac thrombus or vegetation. Magnetic resonance angiography (MRA) of the head and neck without contrast (Figure 2) demonstrated occlusion of the right internal carotid artery (ICA) at its origin without distal reconstitution. Collateral flow directed blood through the anterior communicating artery to the right anterior cerebral artery (ACA) and right middle cerebral artery (MCA). The vertebral arteries were diminutive and the basilar artery was occluded. The posterior communicating arteries and PCAs were not visualized. With this extent of vessel occlusion, even more widespread brain infarction might be expected. However, the complex distribution of the infarcts demonstrated on diffusion-weighted images can be explained by the relative robustness of the patient's leptomeningeal collateral vessels. EEG evaluation demonstrated left parieto-occipital spike wave discharges with associated slowing, excessive subtransients for age, and mild slowing and suppression of background activity.

Bacterial and viral studies were negative. Placental pathology demonstrated a third trimester placenta with acute necrotizing chorioamnionitis and a three-vessel cord with acute funisitis (inflammation of the umbilical cord). Extensive thrombophilia evaluation performed on day of life four (Table 1) failed to identify any inherited or acquired prothrombotic risk factors. However, other than negative DNA mutation analysis, definitive protein abnormalities could not be ruled out until repeat evaluation performed at 3–6 month of life [10]. State metabolic screening through the State of Florida was negative.

Prior to the results of the thrombophilia evaluation, we decided to anticoagulate the patient with LMWH. We based this decision on the occlusion of multiple large blood vessels with evidence of multifocal cerebral infarctions, a pattern indicative of an embolic process. Our rationale for choosing LMWH was to decrease the risk of clot extension, which was suggested by the progressive thrombocytopenia. LMWH was initiated, using a therapeutic antifactor Xa level of 0.5–1.0 IU/mL.

The patient had no further seizure activity, and gradually improved during her admission. The platelet count increased after starting therapy to 156 × 10^3^/mm^3^ and then 253 × 10^3^/mm^3^, at 48 hours and 96 hours, respectively. She was discharged home on day of life 13 from the neonatal intensive care unit taking full oral feedings, and her family was instructed to continue phenobarbital and LMWH with scheduled follow-up.

**Figure 3:**
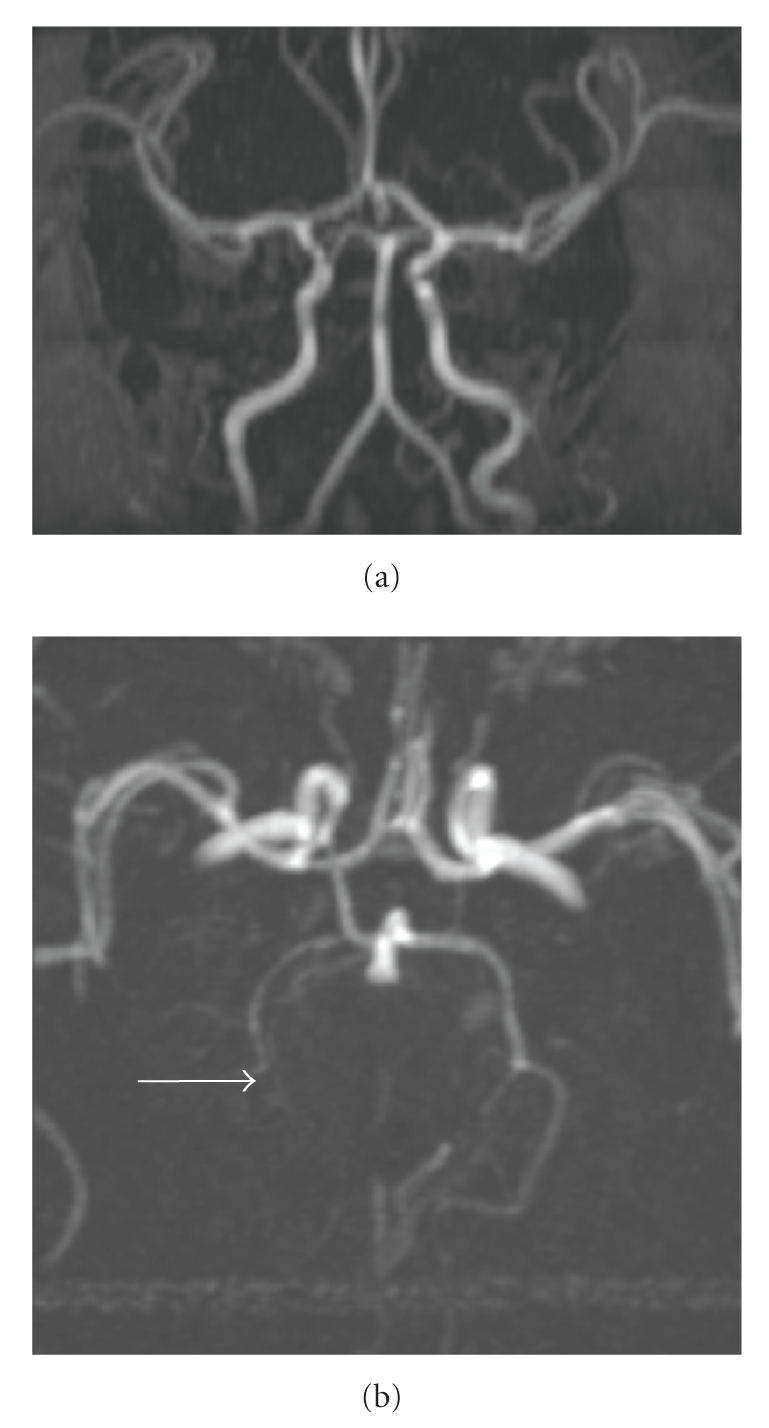
3D MIP images from a noncontrast 2D time of flight MRA demonstrate restoration of flow in the right internal carotid artery and posterior circulation. Residual occlusion of the distal right PCA persists (arrow).

MRI evaluation at four months of age demonstrated bilateral encephalomalacia in areas of prior infarction with no evidence of current or prior hemorrhage. Mild encephalomalacia was also seen in the right PCA territory, which was not ischemic on the initial MRI. This likely reflected an additional infarct which most likely occurred during or soon after the initial scan, possibly before the initiation of LMWH. MRA evaluation (Figure 3) revealed resolution of the right ICA thrombus with overall decreased caliber of the vessel compared to the left, and residual irregularity and narrowing at the superior cervical segment. The intracranial vessels were all patent, although the right PCA was diminutive. Repeat thrombophilia assessment at four months of life was negative (Table 1).

We discontinued LMWH treatment after obtaining the results of the repeat MRI, MRA, and thrombophilia evaluations. Neurological evaluation at 4.5 months of age demonstrated asymmetric tracking and blinking to confrontation which was worse on the left, mildly increased flexor tone of the left upper extremity, normal lower extremity tone and reflexes bilaterally, and age appropriate milestones for gross motor, fine motor, and social interaction. No seizure activity has been observed or suggested on EEG evaluation, and phenobarbital has been weaned. Neuro-ophthalmological examination at 6 months of age demonstrated normal visual acuity for age and normal blinking to confrontation. The patient continues to thrive and develop at home.

## 3. Discussion

Multifocal cerebral infarction is a rare type of perinatal stroke that tends to be embolic in origin [8]. Despite the multiple risk factors ascribed to perinatal stroke, little evidence exists to inform management of these infants after initial stabilization [1, 9]. Previous studies of perinatal stroke have been limited due to small sample size or lack of adequate control groups [6, 8].

Current ACCP guidelines suggest that only neonates with proven cardioembolic arterial ischemic stroke or those with recurrent arterial ischemic stroke should receive treatment with anticoagulation or aspirin therapy [9]. Recommendations from a special writing group of the AHA stroke council and the council on cardiovascular disease in the young [1] suggest that anticoagulation may be considered in neonates with multiple cerebral emboli. Both of these recommendations are based on expert opinion and case series/reports. However, they leave the clinician with difficult treatment decisions to make when faced with a neonate with severe multifocal perinatal thromboembolic stroke (such as our case report).

Recent research has demonstrated increasing evidence that LMWH may play a pivotal role in perinatal stroke [12]. Studies in pediatric patients have shown efficacy and safety of LMWH similar to that observed in adults, and they have demonstrated that LMWH prevents progression of stroke, development of new symptomatic thrombi, and extensions of existing thrombi [12–14]. Punzalan et al. present two children with internal carotid artery thrombi who had either complete or partial resolution of thrombi while being treated with LMWH [13]. One of the largest neonatal studies involving LMWH demonstrated that with careful monitoring and laboratory evidence of therapeutic LMWH effect, complete or partial resolution of thromboembolism was achieved in the majority of cases [15]. Significant bleeding occurred in four of the 62 infants, but none of the events resulted in severe sequelae or death. Other studies and case reports have not only demonstrated similar efficacy, but also decreased clot extension and prevention of embolic phenomenon [16]. One limitation with applying these reports to perinatal stroke is that only four patients in these studies had clots in the central nervous system [15]. Although LMWH does appear to be a safe medication for treatment and prophylaxis of neonates with perinatal stroke, individual judgment (for now) should be applied to specific cases.

This case is characteristic of the severe end of the spectrum of perinatal stroke and emphasizes the difficult acute management decisions regarding such neonates. The comprehensive ACCP guidelines provide recommendations that facilitate the standardized use of antithrombotic and thrombolytic therapy in neonates and children. We appreciate that extreme cases, such as the one presented, are not routinely encompassed by such guidelines, and in these situations optimal treatment may fall outside the recommendations provided. Other sources (such as those of the AHA) can be referred to in order to provide some guidance but the physician may ultimately have to make a balanced decision.

We decided to treat the neonate described above with LMWH based on the severity of the thrombus in the right internal carotid artery and the multifocal nature of the arterial ischemic stroke. Although the probable source of the initial thrombus was the placenta, we believed that there was a high risk for extension of the internal carotid thrombus and/or further embolic complications. We felt that the potential risk associated with bleeding was significantly less than the devastating ramifications of further central nervous system insult. Our patient suffered no complications from the treatment and had resolution of the thrombi, thus supporting the safety and efficacy of LMHW in neonates.

We cannot prove definitively that LMWH resulted in clot resolution without a similar control patient and a timeline involving serial imaging studies. Serial MRA/MRI was not performed due to the extreme cost and the potential morbidity associated with repeated sedation. The patient's thrombocytopenia and clinical condition improved after initiation of LMWH, suggesting that LMWH halted clot extension. Moreover, the follow-up MRI/MRA after four months of LMWH treatment demonstrated almost complete recanalization of the cerebral vessels. This MRI did demonstrate encephalomalacia in the right PCA territory which was not predicted from the original infarct pattern. However, based on consistent clinical improvement, we strongly feel that this infarct occurred in close proximity to the initial MRI, most likely before the initiation of LMWH. In addition, it suggests that LMWH may have prevented further infarcts. Therefore, we feel that this case supports the recommendations provided by the AHA for LMWH treatment in complex cases of multiple cerebral emboli. While we recognize that one case study does not represent strong evidence, we propose that when the ACCP guidelines are again updated, the authors consider review and inclusion of a subset of patients with severe multifocal noncardioembolic stroke who may benefit from anticoagulation. This case also highlights the need for controlled clinical trials to address the management of the broad spectrum of perinatal stroke [2].

## Figures and Tables

**Table 1:** Thrombophilia Evaluation Performed [10, 11].

Test	Performed	Test	Performed
PT	*1 and *2	Anti-B2 glycoprotein IgG	1 and 2
INR	1 and 2	Anti-B2 glycoprotein IgM	1 and 2
aPTT	1 and 2	Anti-phosphatidylserine IgG	1 and 2
Fibrinogen	1 and 2	Anti-phosphatidylserine IgM	1 and 2
Factor V Leiden	1	Heparin cofactor 2	1 and 2
Prothrombin 20210	1	Homocysteine	1 and 2
MTHFR	1	Lipoprotein (A)	1 and 2
Protein C activity	1 and 2	Antithrombin	1 and 2
Protein S activity	1 and 2	Factor VIII activity	2
Anticardiolipin IgG	1 and 2	Lupus anticoagulant	2
Anticardiolipin IgM	1 and 2	Factor XII activity	2
Plasminogen	2		

PT: prothrombin time; aPTT: activated partial thromboplastin time; INR: international normalized ratio; MTHFR: methylenetetrahydrofolate reductase.

*1 Test performed at day of life no. 4; *2 Test performed at four months of age.
